# Overexpression of COP9 signalosome subunits, CSN7A and CSN7B, exerts different effects on adipogenic differentiation

**DOI:** 10.1002/2211-5463.12129

**Published:** 2016-10-11

**Authors:** Xiaohua Huang, Jürgen Ordemann, Johann Pratschke, Wolfgang Dubiel

**Affiliations:** ^1^ Division of Molecular BiologyDepartment of General, Visceral and Transplantational SurgeryCharité – Universitätsmedizin BerlinGermany; ^2^ Department of General, Visceral, Vascular and Thoracic SurgeryCharité – Universitätsmedizin BerlinGermany; ^3^ Department of General, Visceral and Transplantational SurgeryCharité – Universitätsmedizin BerlinGermany

**Keywords:** adipogenesis, COP9 signalosome, CSN7A, CSN7B, LiSa‐2 cells, mouse embryonic fibroblasts

## Abstract

The COP9 signalosome (CSN) is an essential regulator of cullin‐RING‐ubiquitin (Ub) ligases (CRLs), which ubiquitinate important cellular regulators and target them for degradation by the Ub proteasome system (UPS). The CSN exhibits deneddylating activity localized on subunit CSN5, which removes the ubiquitin‐like protein Nedd8 from the cullins of CRLs. CSN‐mediated deneddylation is an important step in the process of CRL remodeling, in which new substrate recognition units are incorporated into Ub ligases to meet changed requirements for proteolysis in cells. For instance, extensive CRL remodeling occurs during adipogenic differentiation when new CRL3s are formed. Diversification of CSN complexes during evolution is most likely another adaptation to meet different cellular requirements. Best known CSN variants are formed by different CSN subunit isoforms. For instance, in plant cells, isoforms have been identified for the MPN‐domain subunits CSN5 (CSN5A and CSN5B) and CSN6 (CSN6A and CSN6B) which form four distinct CSN variants. In mammalian cells CSN^CSN7A^ and CSN^CSN7B^ variants are generated by CSN7 isoforms. We demonstrate that the two variants coexist in human LiSa‐2 cells and in mouse embryonic fibroblasts. During adipogenic differentiation of LiSa‐2 cells CSN7B increases in parallel with an elevation of the total CSN complex. Permanent overexpression of Flag‐CSN7B but not of Flag‐CSN7A accelerates adipogenesis in LiSa‐2 cells indicating a specific function of the CSN^CSN7B^ variant in stimulating adipogenesis. Silencing of CSN7A as well as of CSN7B in LiSa‐2 cells and in mouse embryonic fibroblasts (MEFs) reduces adipogenic differentiation demonstrating that both CSN^CSN7A^ and CSN^CSN7B^ variants are involved in the process.

**Abbreviations**
CAND1: cullin‐associated and neddylation‐dissociated 1
CRL: cullin‐RING‐ubiquitin Ligase
CSN: COP9 signalosome
MEFs: mouse embryonic fibroblasts
MPN: MOV34‐Pad1‐N‐terminal
ORO: Oil‐Red‐O
PCI: proteasome‐COP9 signalosome‐Initiation factor eIF3
SRS: substrate recognition subunit

The mammalian COP9 signalosome (CSN) is a hetero‐octameric complex consisting of subunits CSN1–CSN8 1, 2. As shown by mass spectrometry (MS) 3, 4, cryo‐electron microscopy (cryo‐EM) 5 and crystal structure analysis 6 each complex contains a single copy of each subunit. It is composed of six Proteasome‐COP9 signalosome‐Initiation factor eIF3 (PCI)‐domain subunits including CSN1‐4, CSN7, and CSN8 and two MOV34‐Pad1‐N‐terminal (MPN)‐domain subunits called CSN5 and CSN6.

Increased diversification of the CSN complexes during evolution potentially allowed interactions with different binding partners and substrates as well as diverse expression profiles that meet different cellular requirements. Diversification of CSN variants developed by varying CSN subunit isoforms originated from gene duplication 4, 7, 8, 9, 10 or by alternative translation forms as shown for CSN8 11. For instance, in plant cells isoforms have been identified for the MPN‐domain subunits CSN5 (CSN5A and CSN5B) and CSN6 (CSN6A and CSN6B) 8, 12. In *Arabidopsis*, CSN5A and CSN5B assemble into distinct CSN complexes, whereas CSN^CSN5A^‐containing variant is the dominant form 4, 12. Most importantly, CSN5A‐ and CSN5B‐containing variants play unique roles in plant development 4, 8.

Endogenous CSN^CSN7A^ and CSN^CSN7B^ variants coexist in human red blood cells 1, 3. In other mammalian cells, CSN7A and CSN7B are coexpressed as well 10. CSN7A and CSN7B possess 59% protein sequence identity (see Fig. 1A) 7. According to the NCBI databank, the CSN7A gene is localized on chromosome 12, whereas the CSN7B gene was mapped to chromosome 2. At present, CSN7A and CSN7B expression profiles or CSN^CSN7A^ and CSN^CSN7B^ complex functions are unknown. According to the Genevestigator expression data library CSN7A (COPS7A) and CSN7B (COPS7B) are expressed in all human cells and tissues investigated. The two CSN8 isoforms arise from the alternative translation initiation sites Met1 (CSN8A) or Met6 (CSN8B) 11. The two alternative translation initiation sites and the fact that CSN8A as well as CSN8B are integrated into distinct CSN complexes were confirmed by MS analysis 7.

**Figure 1:**
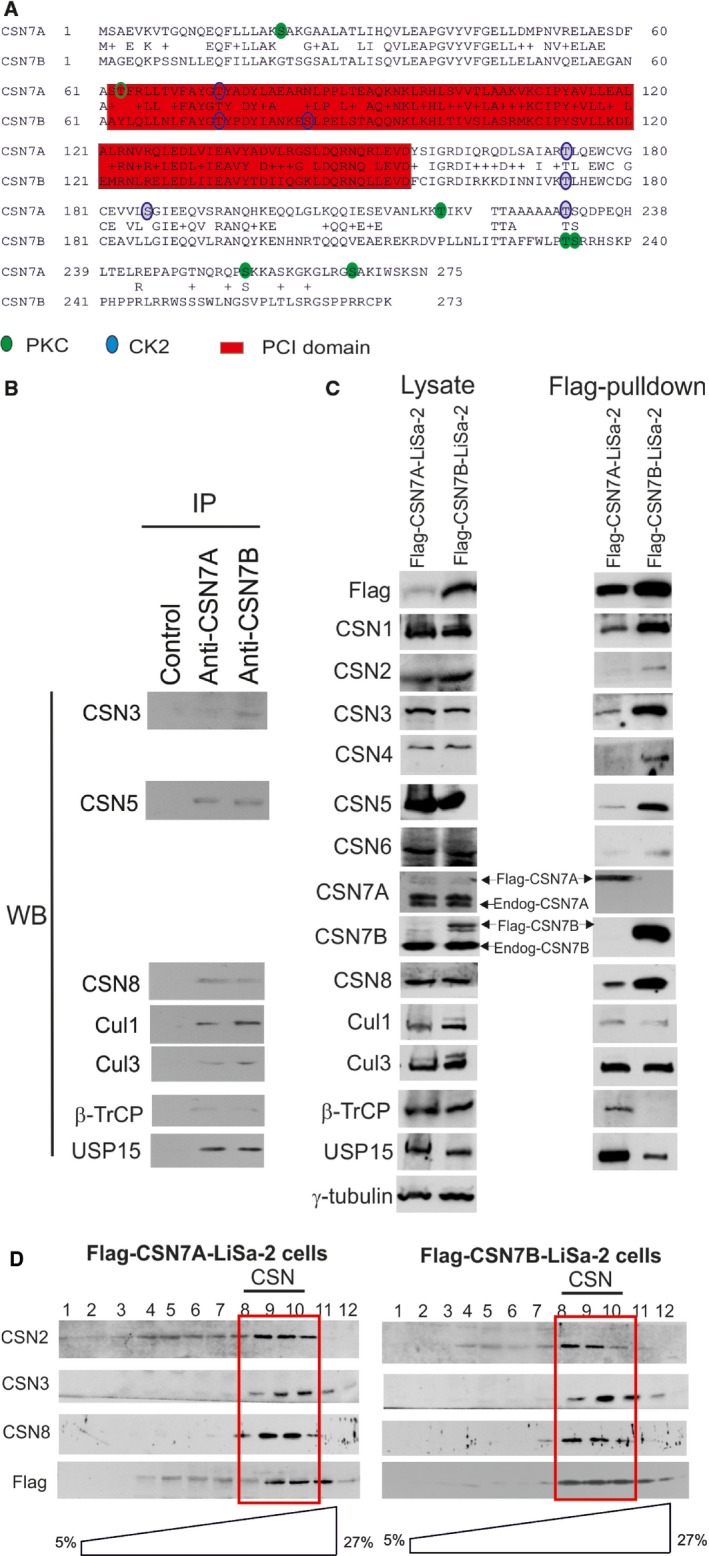
LiSa‐2 cells exhibit distinct CSN^CSN7A^ and CSN^CSN7B^ variants. (A) Sequence alignment of human CSN7A and CSN7B protein. The PCI domains of the two isoforms are localized in the middle of the protein (red). Putative PKC (green) and CK2 (blue) phosphorylation sites are distributed differently throughout the molecules. (B) Immunoprecipitation (IP) with LiSa‐2 cells using specific anti‐CSN7A or CSN7B antibodies and western blotting (WB) using antibodies against selected CSN subunits and CSN‐associated proteins. (C) Lysates of Flag‐CSN7A‐LiSa‐2 cells and of Flag‐CSN7B‐LiSa‐2 cells were analyzed with indicated antibodies by western blotting (left panel). Flag‐pulldowns and subsequent elution by Flag peptide were performed and the eluated proteins were tested with indicated antibodies (right panel). (D) Density gradient centrifugation of lysates from Flag‐CSN7A‐LiSa‐2 cells (left panel) and Flag‐CSN7B‐LiSa‐2 cells (right panel). Western blots were performed with indicated antibodies. As calibrated, under these conditions the CSN peak sedimented into fractions 8, 9, and 10 (red box).

The CSN is a metalloprotease with a MPN+/JAMM motif and, hence, belongs to the JAMM family of deubiquitinating enzymes (DUBs). CSN5 has the catalytic centre and it needs structural rearrangements in the CSN holo complex for its activation 6, 13. It is responsible for the CSN‐mediated removal of Nedd8 from cullins called deneddylation 14. The CSN is highly specific for neddylated cullin as substrate 5, 15.

The CSN forms supercomplexes with cullin‐RING ubiquitin (Ub) ligases (CRLs) comprising the largest family of Ub ligases with more than 250 members 16, 17. The cullin family (Cul1‐7) provides the scaffold for CRL complexes. The C‐terminal part of the cullins is associated with the RING H2 finger proteins, Rbx1 or Rbx2, which bind the E2 with the activated Ub. Substrate‐adaptor proteins (Skp1, elongin C, or DDB1) are associated with the N‐terminal part of cullins. They recruit substrate recognition subunits (SRSs) to the complex 18 such as F‐box proteins (FBP) in case of Cul1 complexes (CRL1) and BTB proteins for CRL3s 17, 19, 20. Deneddylation by the CSN protects CRL components from autoubiquitination 21. In addition, it is a first step in the remodeling of CRLs, an adaptation to changed substrate specificity requirements in cells. The Cullin‐Associated and Neddylation‐Dissociated 1 (CAND1) binds to deneddylated cullins and acts as a protein exchange factor for CRL1^FBP^. It augments the dissociation of Skp1‐FBP modules by million‐fold 22. This allows the recruitment of a new FBP to Cul1. Depletion of CAND1 in *Schizosaccaromyces pombe* 23, in *Saccharomyces cerevisiae* 24 or in human cells 22, 25 profoundly alters the cellular repertoire of CRL1 complexes. Recently we have shown that CAND1 is an exchange factor for CRL3s as well 26.

Together with the ubiquitin proteasome system (UPS), the CSN is responsible for cell cycle progression 27, DNA repair 27, 28, cell differentiation 29, 30, 31, development 2, 29, 31, and tumorigenesis 32, 33. More recently we have shown that the CSN has a function in adipocyte differentiation 34. Adipogenesis is maintained by an interplay between essential transcription factors such as peroxisome proliferator‐activated receptor γ (PPAR‐γ), the CCAAT/enhancer‐binding proteins α and β (CC/EBPα and β), and their antagonist CCAAT enhancer‐binding homologous protein (CHOP). Most of the key regulators of adipogenesis are targeted by the UPS 35, 36. CHOP is ubiquitinated via CRL3 containing the SRS Keap1 34, which is integrated into CRL3 complexes in a CAND1‐dependent manner 26. Permanent downregulation of CSN1 subunit leads to an increase in CHOP and completely blocks adipogenesis 34 demonstrating a pivotal role for the CSN in adipocyte differentiation.

At the moment it is unclear whether CSN^CSN7A^ and CSN^CSN7B^ variants possess different characteristics including altered affinities to CSN‐associated proteins such as CRLs. Moreover, it is obscure whether CSN^CSN7A^ or CSN^CSN7B^ or both are necessary for adipogenesis. Here, we show that CSN^CSN7A^ and CSN^CSN7B^ variants coexist in human LiSa‐2 preadipocytes. While overexpression of CSN7A or CSN7B caused different effects on adipogenic differentiation in LiSa‐2 cells, downregulation of CSN7A as well as CSN7B led to retardation of adipogenesis.

## Results and Discussion

### In LiSa‐2 cells distinct CSN^CSN7A^ and CSN^CSN7B^ variants coexist

Using specific antibodies for CSN7A and CSN7B endogenous CSN complexes were immunoprecipitated from LiSa‐2 cell lysates (see Fig. 1B). Selected CSN subunits were detected by western blotting in the precipitates indicating the existence of distinct CSN7A‐ as well as CSN7B‐containing complexes. In addition, the CSN‐associated proteins Cul1, Cul3, the F‐box protein β‐TrCP, and the ubiquitin‐specific protease 15 (USP15) were identified in the same precipitates (Fig. 1B). To address the question about the composition and interactions of CSN^CSN7A^ and CSN^CSN7B^ complexes, we established LiSa‐2 cell lines that stably express Flag‐CSN7A or Flag‐CSN7B. This method has been successfully applied with permanently expressed Flag‐CSN2 in mouse B8 fibroblasts in which approximately 50% of endogenous CSN incorporated Flag‐CSN2 37. Moreover, recently we have shown that Flag‐pulldowns are proper alternatives for CSN isolation 5 compared to the time consuming CSN preparation from red blood cells 38. Flag‐CSN7A‐LiSa‐2 and Flag‐CSN7B‐LiSa‐2 cells were tested by western blotting (Fig. 1C, left panel). Specific anti‐CSN7A and anti‐CSN7B antibodies were used to detect endogenous CSN7A and CSN7B and overexpressed Flag‐CSN7A and Flag‐CSN7B in LiSa‐2 cells. As seen in Fig. 1C, left panel (Lysate) the expression of Flag‐CSN7A in Flag‐CSN7A‐LiSa‐2 cells and the expression of Flag‐CSN7B in Flag‐CSN7B‐LiSa‐2 cells amounted to about 20–30% of endogenous CSN7A or CSN7B, respectively. Data obtained with LiSa‐2 cell lysate (Fig. 1C, left panel) clearly show a coexpression of endogenous CSN7A and CSN7B in these cells. It seems that CSN7A is less expressed as compared to CSN7B. On the other hand, the anti‐CSN7A antibody seems to be less efficient as compared to the anti‐CSN7B antibody. The anti‐Flag‐antibody showed a nice reaction with Flag‐CSN7A as well as with Flag‐CSN7B, indicating reduced expression of Flag‐CSN7A as compared to Flag‐CSN7B in LiSa‐2 cells.

Cells were lysed and Flag‐pulldowns were performed as described 37. In Fig. 1C, right panel, all eight CSN subunits were detected in CSN^Flag‐CSN7A^ as well as in CSN^Flag‐CSN7B^ complexes. Using specific anti‐CSN7A and anti‐CSN7B antibodies revealed that no CSN7B was detected in CSN^Flag‐CSN7A^ and no CSN7A in CSN^Flag‐CSN7B^ pulldowns (Fig. 1C, right panel). Interestingly, Flag‐pulldowns indicated that relatively more USP15, β‐TrCP, Cul1, and Cul3 were associated with CSN^Flag‐CSN7A^ as compared to CSN^Flag‐CSN7B^ complexes.

Density gradient centrifugation as shown in Fig. 1D was performed to check the integration of Flag‐tagged CSN7A and CSN7B into endogenous CSN complexes in Flag‐CSN7A‐LiSa‐2 and in Flag‐CSN7B‐LiSa‐2 cells. As visualized by the anti‐Flag‐antibody, the majority of both Flag‐CSN7A and Flag‐CSN7B sedimented into the peak fractions of the CSN.

### Overexpression of Flag‐CSN7B, but not of Flag‐CSN7A, accelerates adipogenic differentiation in LiSa‐2 cells

**Figure 2:**
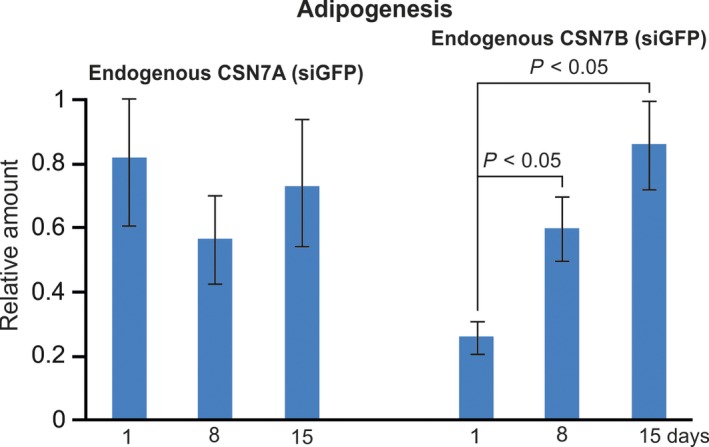
Dynamics of endogenous CSN7A and CSN7B expression levels during adipogenesis. LiSa‐2 cells treated with siGFP were stimulated for adipogenesis using a hormone cocktail and after 1, 8, and 15 days cells were lysed for analysis. Western blots of endogenous CSN7A and CSN7B data from control cells (siGFP cells) as shown in Fig. 4A were quantified by densitometry. CSN7A occurs as a doublet. Based on the molecular mass the lower band was used for calculation. Results were normalized to γ‐tubulin and calculated as relative amount per day of adipogenesis. Data of three independent experiments are expressed in means ± SD. Statistical significance is indicated (*P* < 0.05).

During LiSa‐2 cell adipogenesis the expression of CSN subunits increases 26. Permanent downregulation of CSN expression blocks adipogenesis 34. To estimate relative amounts of endogenous CSN7A and CSN7B protein in control cells (siGFP cells) during adipogenesis we used data from three experiments. LiSa‐2 cells treated with siGFP, siCSN7A, and siCSN7B were stimulated for adipogenesis (see Fig. 4A) using a hormone cocktail consisting of insulin, triiodthyronine, and cortisol as described before 34, 39. After 1, 8, and 15 days, cells were lysed and lysates were probed with indicated antibodies. Bands obtained from controls (siGFP) as presented in Fig. 4A were determined by densitometry and normalized against γ‐tubulin. As shown in Fig. 2, at the beginning of adipogenesis (day 1) CSN7A protein levels were higher as compared to CSN7B. However, during adipogenesis CSN7B expression level increased significantly reaching the expression levels of CSN7A on day 8 and 15, whereas CSN7A expression did not change significantly.

**Figure 3:**
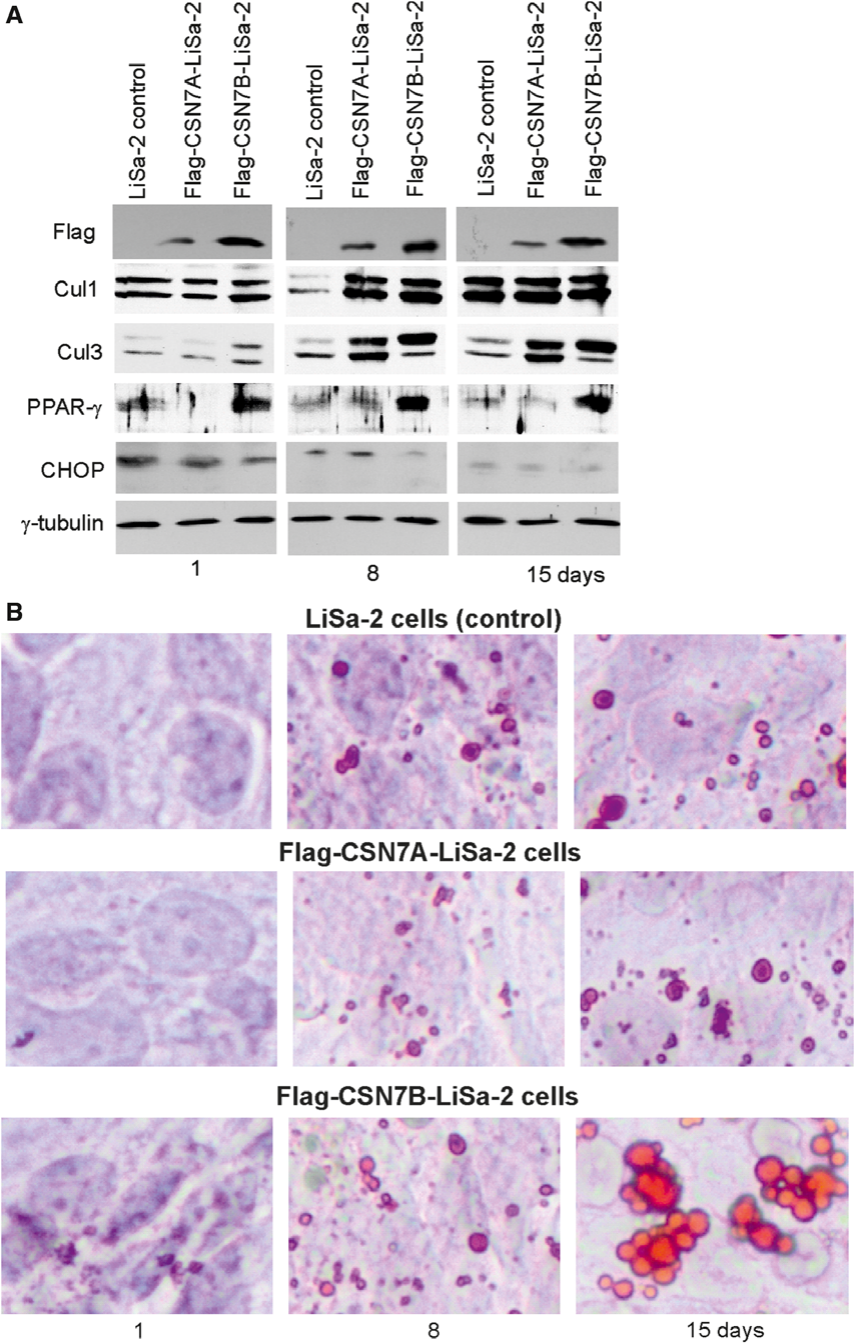
Dynamics of Cul1, Cul3, PPAR‐γ, and CHOP expression during adipogenesis in response to Flag‐CSN7A or Flag‐CSN7B overexpression in LiSa‐2 cells. (A) Western blotting with lysates from LiSa‐2 cells (controls), Flag‐CSN7A‐LiSa‐2 cells and Flag‐CSN7B‐LiSa‐2 cells using indicated antibodies. After stimulation of adipogenesis cells were lysed at day 1, 8, and 15 and analyzed by western blotting. (B) Lipid droplet formation was visualized by staining with ORO and light microscopy after day 1, 8, and 15 of adipogenic differentiation.

The western blot in Fig. 3A shows the impact of permanent overexpression of Flag‐CSN7A or Flag‐CSN7B on regulators of adipogenesis. PPAR‐γ is a master regulator of adipogenesis which is also characterized by an increase in neddylated Cul3 26 and by downregulation of CHOP 34. The anti‐Flag antibody indicates the overexpression of Flag‐CSN7A or Flag‐CSN7B. Figure 3A clearly demonstrates that overexpression of Flag‐CSN7B caused an induction of PPAR‐γ expression, an early accumulation of neddylated Cul3 as well as a downregulation of CHOP. This parallels with an increased formation of lipid droplets (LD) as visualized by Oil‐Red‐O (ORO) staining (Fig. 3B). LiSa‐2 cells permanently expressing Flag‐CSN7B showed a significantly accelerated adipogenesis in comparison to Flag‐CSN7A‐LiSa‐2 and control cells.

### Silencing of CSN7A and CSN7B retards adipogenesis in LiSa‐2 cells as well as in mouse embryonic fibroblasts

**Figure 4:**
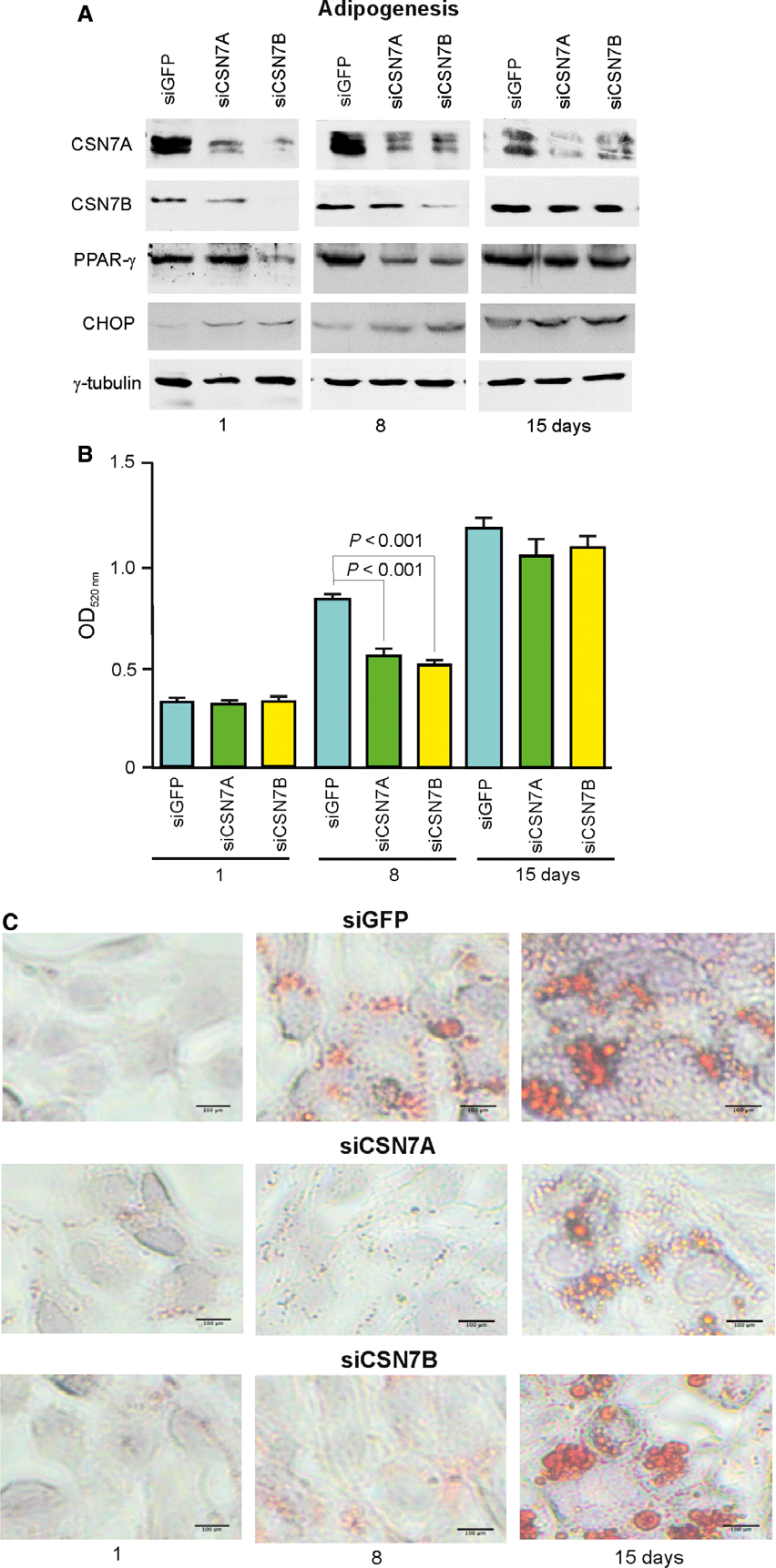
Transient silencing of CSN7A or CSN7B during adipogenesis in LiSa‐2 cells. LiSa‐2 cells were transiently transfected with siRNA against CSN7A (siCSN7A) or CSN7B (siCSN7B). (A) Adipogenesis was induced and after indicated times western blots were performed with indicated antibodies. (B) LiSa‐2 cells transiently transfected with siRNA against CSN7A or CSN7B were stained with ORO, which was quantified by photometry (see Materials and Methods). Data obtained from four independent experiments are expressed as means ± SEM. Statistical significance is indicated. (C) Lipid droplet formation was visualized by staining with ORO and light microscopy in control cells (siGFP) and LiSa‐2 cells transfected with siRNA against CSN7A (siCSN7A) and CSN7B (siCSN7B).

To investigate the impact of CSN7A and CSN7B on the dynamics of adipogenesis regulators, we transiently downregulated CSN7A or CSN7B with specific siRNA during the differentiation of LiSa‐2 cells. As shown in the western blot in Fig. 4A, downregulation of CSN7A as well as CSN7B led to a reduction in PPAR‐γ and an increase in CHOP after 1 and 8 days of differentiation. As expected, after 15 days of differentiation, the effect of transient transfection was neutralized and protein concentrations were normalized. ORO staining was performed to quantify lipid production during adipogenesis. As demonstrated in Fig. 4B, there was a significant reduction of lipid production in response to CSN7A as well as CSN7B downregulation after 8 days of differentiation. The corresponding microscopy in Fig. 4C shows retarded LD production upon silencing of both CSN7A and CSN7B.

**Figure 5:**
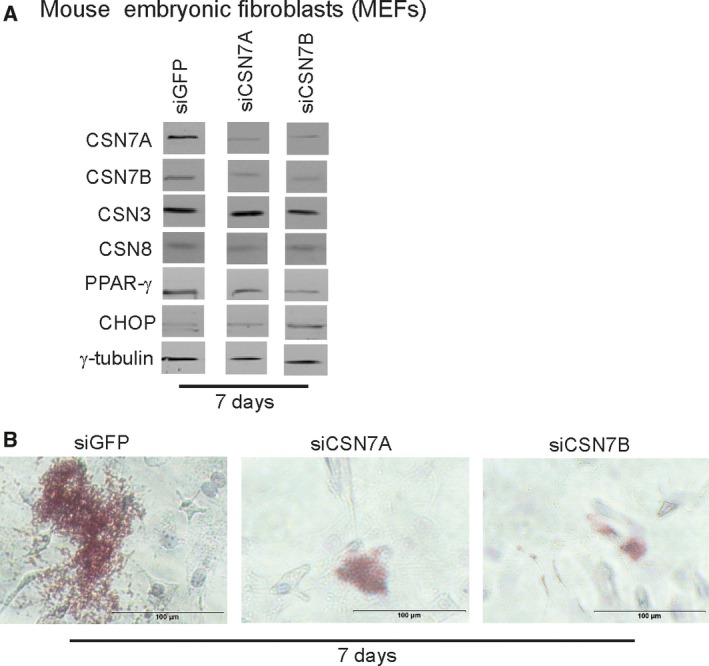
Transient silencing of CSN7A and CSN7B during adipogenesis in MEFs. (A) Adipogenesis was induced by a hormone cocktail (see Materials and Methods). After 7 days of differentiation cells were lysed and analyzed by western blotting using the indicated antibodies. (B) Lipid droplet formation was visualized by staining with ORO and light microscopy in control MEFs (siGFP) and MEFs transiently transfected with siRNA against mouse CSN7A (siCSN7A) and CSN7B (siCSN7B).

To confirm the effect of CSN7A and CSN7B silencing on adipogenesis in another model system for adipogenic differentiation we used mouse embryonic fibroblasts (MEFs) 40. Using a specific hormone cocktail (see Materials and Methods) adipogenic differentiation can be induced in MEFs. Figure 5 summarizes data obtained after 7 days differentiation of MEFs. Similar as in LiSa‐2 cells, downregulation of CSN7A as well as of CSN7B reduced the expression of PPAR‐γ and increased the level of CHOP as compared to siGFP controls (Fig. 5A). Likewise, as demonstrated in Fig. 5B, ORO staining revealed a retardation of LD formation after transient silencing of CSN7A as well as CSN7B on day 7 of MEF differentiation. Unfortunately downregulation of CSN7A led to accompanied reduction of CSN7B and vice versa. At the moment, the reason for this effect is unclear.

### Acceleration of adipogenesis in Flag‐CSN7B‐LiSa‐2 cells specifically depends on the increase of CSN7B

**Figure 6:**
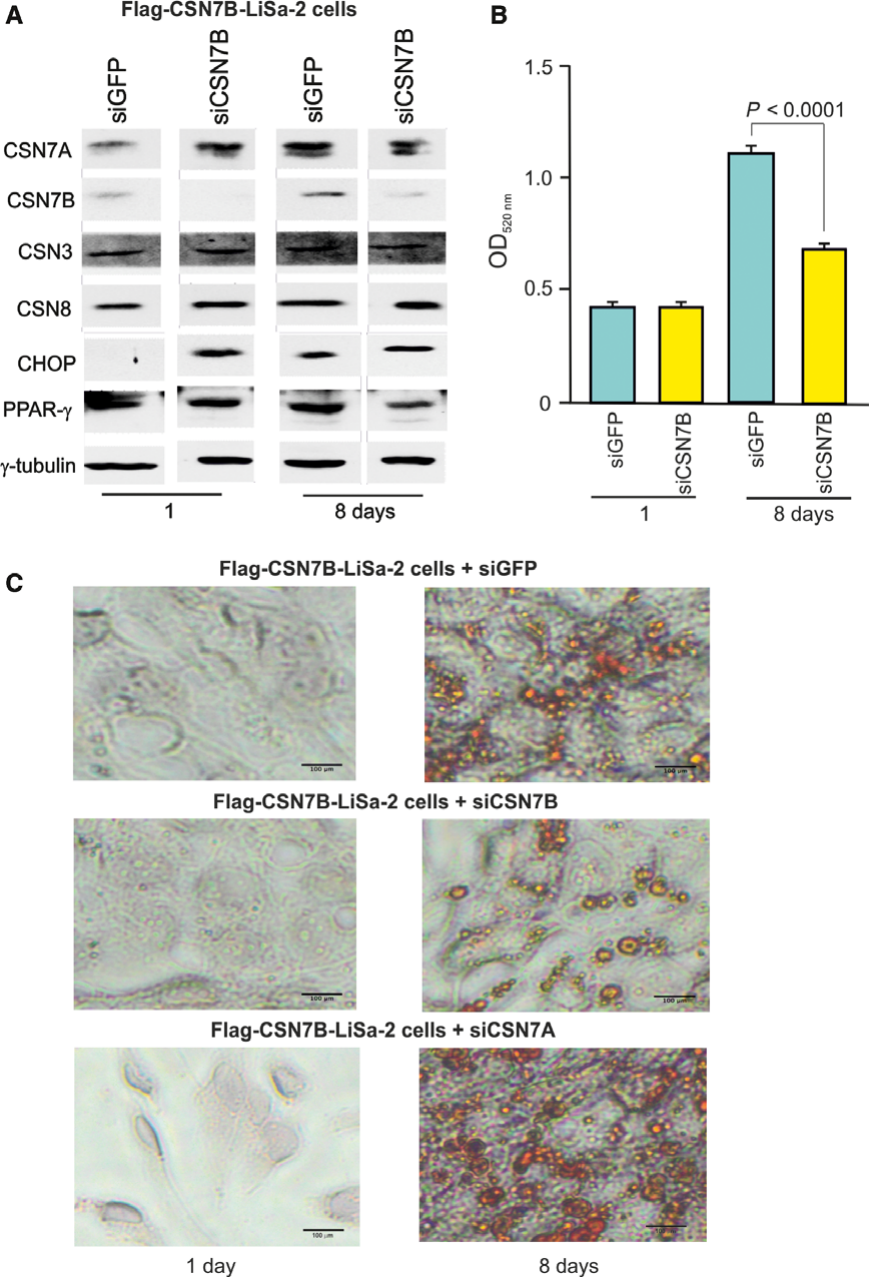
Transient silencing of CSN7B in Flag‐CSN7B‐LiSa‐2 cells during adipogenesis. Flag‐CSN7B‐LiSa‐2 cells were transiently transfected with siRNA against GFP (control), CSN7B, and CSN7A. (A) Adipogenesis was induced and after indicated times western blots were performed with indicated antibodies. (B) Transient silencing of CSN7B in Flag‐CSN7B‐LiSa‐2 cells during adipogenesis. ORO staining was quantified by photometry after 1 and 8 days of differentiation. Data obtained from four independent experiments are expressed as means ± SEM. Statistical significance is indicated. (C) Flag‐CSN7B‐LiSa‐2 cells were transiently transfected with siRNA against GFP (control), CSN7B, and CSN7A. Adipogenesis was induced and after indicated times ORO staining was performed and lipid droplet formation was visualized by light microscopy.

LiSa‐2 cells stably expressing Flag‐CSN7B resulting in accelerated adipogenesis were transfected with specific siRNA against CSN7B. Again, adipogenesis was stimulated by the hormone cocktail as described above. Cells were analyzed by western blotting and ORO staining after 1 and 8 days of differentiation. As shown in Fig. 6A there were high concentrations of PPAR‐γ and low of CHOP in Flag‐CSN7B‐LiSa‐2 cells in the absence of specific siRNA as it was demonstrated before (see Fig. 3A). In the presence of siCSN7B, PPAR‐γ was reduced and CHOP increased after 1 and 8 days of differentiation. This was accompanied by significantly reduced LD formation as demonstrated by ORO staining and its quantification in Fig. 6B, C, respectively. Transfection of Flag‐CSN7B‐LiSa‐2 cells with siCSN7A did not affect accelerated adipogenesis in these cells. As demonstrated by ORO staining in Fig. 6C in the presence of siCSN7A the acceleration of adipogenesis in Flag‐CSN7B‐LiSa‐2 cells was as high as in the siGFP control. These results show that the acceleration of adipogenesis in Flag‐CSN7B‐LiSa‐2 cells most likely depends on CSN7B expression.

## Conclusions

In human LiSa‐2 cells at least two CSN variants coexist, CSN^CSN7A^ and CSN^CSN7B^. We provide evidence that the two CSN variants might have different functions in adipogenic differentiation. Although endogenous CSN7B increases during adipogenesis, there is no change in endogenous CSN7A (Fig. 2). Increased expression of CSN7B is accompanied with an increase of the CSN complex during adipogenic differentiation 26 indicating a unique role of the CSN^CSN7B^ variant in adipogenesis. Moreover, overexpression of CSN7B, but not that of CSN7A, accelerates adipogenesis. These effects can be explained by a specific function of CSN^CSN7B^ variant in stimulating adipogenesis. Perhaps CSN^CSN7B^ preferentially binds to certain CRLs or has a modified deneddylating activity and this promotes adipogenesis. Recently it has been shown that CSN7 might be involved in CSN–CRL interaction leading to induced‐fit CSN activation and regulation of CRLs 41. This suggestion is confirmed by the observation that CSN^CSN7A^ and CSN^CSN7B^ variants seem to bind β‐TrCP and USP15 with different affinities in Flag‐pulldowns (Fig. 1B).

On the other hand, downregulation of CSN7A as well as CSN7B slowed down adipogenesis indicating that both CSN variants are necessary for adipogenesis. Future experiments are necessary to reveal the underlying mechanism of CSN7A and CSN7B effects on adipogenesis.

## Materials and methods

### Cell culture and transfection

Human liposarcoma LiSa‐2 cells (a gift from Wabitsch and coworkers 39) were used as an adipocyte differentiation model growing in ISCOVE/RPMI (4 : 1) medium containing 10% fetal calf serum, 100 U·mL^−1^ penicillin, and 0.1 mg·mL^−1^ streptomycin as described 39. Differentiation was induced by culturing cells in serum‐free medium with supplements including 1 nm insulin, 20 pm triiodthyronine, and 1 mm cortisol according to a protocol by Wabitsch *et al*. 39. During differentiation the medium was changed every 2 or 3 days. Differentiation and formation of lipids was monitored by ORO staining and light microscopy, whereby nuclei were visualized with hemotoxylin 34. ORO was quantified according to published protocol 42. In brief, after washing cells four times in PBS, the ORO‐stained lipids were extracted with 4% NP‐40 and 96% isopropanol. The supernatants were measured spectrophotometrically at 520 nm.

Differentiated cells were harvested on different days of differentiation as indicated and lysed with ice‐cold triple‐detergent lysis buffer (50 mm Tris‐HCl, pH 8.5; 150 mm NaCl; 0.02% (w/v) sodium azide; 0.1% (w/v) SDS; 1% (v/v) NP‐40; 0.5% (w/v) sodium deoxycholate) or single‐detergent lysis buffer (50 mm Tris‐HCl, pH 7.4; 150 mm NaCl; 1 mm EDTA, pH 8.0; 1% Triton X‐100) with freshly added PMSF (1 mg·mL^−1^) and aprotinin (10 μg·mL^−1^).

Mouse embryonic fibroblasts were retrieved from disaggregated embryos at embryonic day (E) 12.5–13.5 (gift from Klaus‐Peter Knobeloch) and are a standard cell model for studying adipogenesis *in vitro* 40. MEFs were cultured at 37 °C and 5% CO_2_ in normal MEF culture medium and adipogenic differentiation was induced by change of culture medium to MEF differentiation medium. The MEF culture medium consisted of high glucose DulbecoMEM, 10% FCS, 100 U·mL^−1^ penicillin, 0.1 mg·mL^−1^ streptomycin, 0.2 mm glutamine, 0.1 mm β‐mercaptoethanol, and 0.2 mm nonessential amino acids. The MEF differentiation medium was the culture medium supplemented with 0.5 mm methylisobutylxanthine, 1 μm dexamethasone, 10 μg·mL^−1^ insulin, and 10 μm troglitazone.

### Stable transfections with Flag‐CSN7A and Flag‐CSN7B and transient transfections with siCSN7A and siCSN7B

For stable transfection, CSN7A or CSN7B cDNA were obtained by PCR and cloned into pCMV‐3Tag‐1a vector (Stratagene, La Jolla, CA, USA) encoding 3 N‐terminal Flag‐tags. These plasmids were transfected into LiSa‐2 cells. Single clones were used to obtain resistant cell lines with 0.25 mg·mL^−1^ of neomycin.

For transient transfection, human siCSN7A, siCSN7B and siGFP (Cell Signaling, Denver, CO, USA) were transfected in LiSa‐2 cells or mouse siCSN7A, siCSN7B and siGFP (Santa Cruz, Santa Cruz, CA, USA) were transfected in MEF cells. After 48 h, the adipogenic differentiation of LiSa‐2 cells was induced by changing the culture medium (see above). LiSa‐2 cells were then harvested for western blot analysis and for ORO staining at day 1, 8, and 15 of differentiation. For MEFs, adipogenic differentiation was induced by a change in culture medium (see above) for 4 days, after which cells were cultured in normal medium for another 3 days (total of 7 days) 43 and harvested for western blotting and for ORO staining.

### Immunoprecipitation, Flag‐pulldowns, glycerol gradient, western blotting, and densitometry

Immunoprecipitations with the specific antibodies against CSN7A (Santa Cruz) and CSN7B (Santa Cruz) were carried out as stated earlier 44. Flag‐pulldowns using Flag‐CSN7A‐LiSa‐2 and Flag‐CSN7B‐LiSa‐2 cells were outlined before 37. Just in brief, lysates were loaded onto the prepared ANTI‐FLAG M2 affinity column (Sigma, St. Louis, MO, USA). After washing with 20 column volumes of 1× TBS (50 mm Tris‐HCl, pH 7.4, 150 mm NaCl, 1 mm EDTA), proteins were eluted by competition with the Flag peptide (100 μg·mL^−1^) as recommended by the manufacturer (Sigma). Eluted proteins were used for western blots.

Glycerol gradient centrifugation analysis was performed as described 34. Fractions with different density (5–27% glycerol) were separated on SDS/PAGE and analyzed by western blotting. Density gradient was standardized with purified human CSN (about 400 kDa, fractions 8–10). Western blots were carried out using antibodies against Flag (Sigma), CSN1, CSN2 (ENZO, New York, NY, USA), CSN3 45, CSN4 (ENZO), CSN5, CSN6, CSN7A, CSN7B (Gene Tex, Irvine, CA, USA), CSN8 (ENZO), Cul1 (CBD Biosciences, Franklin Lakes, NJ, USA), Cul3 (CBD Biosciences), β‐TrCP (Santa Cruz), USP15 (Abcam, Cambridge, UK), PPAR‐γ, CHOP, and γ‐tubulin (Santa Cruz).

Densitometry was carried out using imagej software (NIH). Statistics were calculated with graphpad instat3 (Santiago, MN, USA). Error bars are standard deviations (SD) or standard error of the mean (SEM) and unpaired Student's *t*‐test was applied for statistical analysis.

## Author contributions

XH performed most of the experiments, JO designed this study. JP gave helpful suggestions on the manuscript. WD designed the study and wrote the manuscript.
